# A Rare Recombination in the *Phytophthora sojae* Genome Allows the Redefinition of a Major Avirulence Gene

**DOI:** 10.1111/mpp.70326

**Published:** 2026-08-13

**Authors:** Yanick Asselin, Adèle Deshaies, Caroline Labbé, Vanessa Tremblay, François Belzile, Richard Bélanger

**Affiliations:** ^1^ Département de Phytologie Université Laval Québec Québec Canada; ^2^ Institut de Biologie Intégrative et Des Systèmes (IBIS) Université Laval Québec Québec Canada

**Keywords:** *Avr3c*, *Avr4/6*, effector, *Glycine max* (L.) Merr, *Rps3c*, *Rps4*, *Rps6*

## Abstract

Significant yield losses in soybean production are imputable to Phytophthora root and stem rot (PRR) caused by *Phytophthora sojae*. Resistance to PRR relies on resistance genes (*Rps*) that recognize key effectors from the pathogen, which are encoded by avirulence genes (*Avr*). Key *Avr* haplotypes can be monitored in natural *P. sojae* populations and exploited to predict which resistance gene can be deployed to provide protection to the soybean crop. In this study, we reassessed the identity of the avirulence gene whose protein product is recognized by *Rps6*. Following extensive soil sampling and single‐spore isolation of a large population of *P. sojae*, we found two salient isolates carrying a rare recombination between the closely linked effectors *Avr3c* and *Avr4/6*. Using a PCR assay and molecular markers, we showed that only allelic variations at the *Avr3c* locus were in perfect association with the phenotypes of the isolates on *Rps3c*, *Rps4* and Rps6 resistance. Consistent with these findings, a soybean protoplast cell‐death assay further showed that only *Avr3c*, and not *Avr4/6*, triggered *Rps6*‐dependent recognition. Furthermore, whole‐genome resequencing and *de novo* assembly of the two isolates revealed the full extent of this genomic rearrangement. Together, these results establish *Avr3c* as the bona fide avirulence gene corresponding to *Rps6* and offer a more reliable target for the pathotyping of *P. sojae*, which ultimately leads to a better deployment of resistant soybean cultivars.

## Graphical abstract

Reassessment of the identity of the avirulence gene corresponding to the soybean resistance gene *Rps6*. From a large natural population of *Phytophthora sojae*, a rare recombination between two effectors was found and investigated.

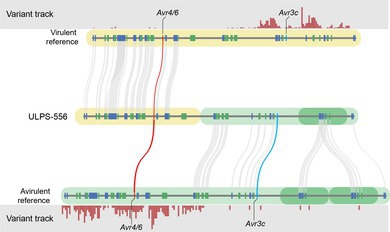

1

*Phytophthora sojae*, the causal agent of Phytophthora root and stem rot (PRR), is one of the 10 most important oomycete plant pathogens (Kamoun et al. 2015). It caused about $200 million of yield losses in the United States alone in 2024 (Crop Protection Network 2025). Management of PRR relies predominantly on the use of genetic control where major 'resistance to *Phytophthora sojae'* (*Rps*) genes offer reliable protection from germination to harvest. Over 40 *Rps* genes and alleles have been reported (Chen et al. 2025) but only six are commercially exploited: *Rps1a*, *Rps1b*, *Rps1c*, *Rps1k*, *Rps3a* and *Rps6*. For the optimal control of PRR, the deployment of an *Rps* gene must coincide with the *P. sojae* pathotypes present in a field; therefore the specificity of each resistance gene must be precisely known. From the 456 RxLR effector genes revealed by the recent telomere‐to‐telomere assembly of the *P. sojae* genome (Zhang et al. 2024), only a few selected members are recognized by the product of an *Rps* gene; these include *Avr1a* (Qutob et al. 2009), *Avr1b* (Shan et al. 2004), *Avr1c* (Na et al. 2014), *Avr1d* (Na et al. 2013), *Avr1k* (Song et al. 2013), *Avr3a/5/8* (Dong, Yu, et al. 2011; Qutob et al. 2009; Arsenault‐Labrecque et al. 2022), *Avr3b*/*11* (Dong, Yin, et al. 2011; Asselin et al. 2025), *Avr3c* (Dong et al. 2009) and *Avr4/6* (Dou et al. 2010). Distinct SNP haplotypes have been associated with alleles of each *Avr* gene to predict the response to infection of resistant soybean lines (Arsenault‐Labrecque et al. 2018). The near‐perfect association between *Rps* genes and their corresponding effectors makes this pathosystem a salient example of the gene‐for‐gene concept based on effector‐triggered immunity (ETI).

Despite their initial distinction, *Rps4* and *Rps6* were both reported to recognize *Avr4* and *Avr6*, two avirulence genes originally described as tightly linked and later redefined as a single locus, *Avr4/6*, because of the complete cosegregation of the traits (Gijzen et al. 1996; Whisson et al. 2004). Subsequently, Dou et al. (2010) produced crosses between *P. sojae* isolates, discriminant for their virulence against *Rps4* and *Rps6*, which led to the identification of an effector, *Avh171*, on chromosome 5 (Chr5) from the Zhang et al. (2024) genome assembly in a region close to the *Rps3c* corresponding effector, *Avr3c* (Dong et al. 2009; Dou et al. 2010). Transient expression assays further demonstrated that both *Avr4/6* and *Avr3c* can trigger cell death in resistant soybean lines. However, the recent description and cloning of *Rps6* showed that it is functionally redundant with *Rps3c* and *Rps4* (Asselin et al. 2026). Indeed, all three *Rps* genes were shown to confer resistance through recognition of *Avr3c*. To reconcile these contradictory publications, we present here evidence of a rare recombination event between the two closely linked genes coding for these effectors, *Avr3c* and *Avr4/6*. PCR‐based genotyping was employed to show that only allelic variations at the *Avr3c* locus were associated with virulence towards *Rps3c*, *Rps4* and *Rps6*. A cell death assay in soybean protoplasts further confirmed the sole interaction of *Avr3c* with *Rps6*. Moreover, long‐read whole‐genome resequencing and *de novo* genome assembly uncover the genomic rearrangement underlying the reassignment of a major avirulence determinant, thereby redefining the genetic basis of *Rps3c*‐, *Rps4*‐ and *Rps6*‐mediated resistance in *P. sojae*.

During a three‐year survey conducted in five Canadian provinces, soil samples were collected in soybean fields with a history of PRR to study the virulence profiles of natural *P. sojae* populations. Single‐spore isolates were recovered from soil samples according to the baiting method described by Tremblay et al. (2021). A collection of 236 isolates was retrieved and fully pathotyped using a differential of 13 resistant soybean near‐isogenic lines (NILs) and one susceptible control in a hydroponic assay (Lebreton et al. 2018). All isolates were virulent on the susceptible control and showed a wide range of pathotypes (Table S1). As shown in Figure 1, the most prevalent pathotypes were 1a (98%), 1c (96%) and 7 (94%), closely followed by 1b (71%), 1d (80%) and 3a (69%). In contrast, the least prevalent pathotypes were 3b/11 (9%) and 3c/4/6 (13%), making *Rps3b/11* and *Rps6* the most promising resistance genes to be deployed in Canada. Particular attention was given to the phenotypes of *Rps3c*, *Rps4* and *Rps6* as these resistance genes were recently shown to be redundant (Asselin et al. 2026). The isolates should either display virulence or avirulence towards all three resistant lines, with no discordant phenotypes. Consistent with this expectation, 31 of the 236 isolates were capable of infecting all *Rps3c*, *Rps4* and *Rps6* resistant soybean lines, corresponding to a frequency of 13.1% for this pathotype. No isolate was virulent on only one or two of the three soybean NILs. All remaining isolates (205) were avirulent to these same lines but virulent against other resistant lines. Then, to assess their genotype at the *Avr3c* and *Avr4/6* loci, all 236 isolates were grown in pure cultures on V8 agar covered by cellulose to facilitate the harvest of mycelium. Thereafter, samples were homogenized and DNA was extracted using a modified CTAB method. Using the sequences of *Avr3c* reported by Huang et al. (2019) (Figure S1), PCR primers were designed to discriminate between virulent and avirulent alleles. For *Avr4/6*, the coding region of the virulent and avirulent alleles is identical (Figure S1), with polymorphism confined to the 5′ untranslated region and further upstream of the gene (Dou et al. 2010). Consequently, a 15‐bp deletion located 524 bp upstream of the gene and previously described by Arsenault‐Labrecque et al. (2018) was exploited to distinguish the two alleles (Table S2). All 236 isolates were then genotyped for their alleles of *Avr3c* and *Avr4*/6 in a PCR assay. For 234 *P. sojae* strains, the genotypes of both genes were perfectly concordant with their phenotypes. In contrast, two isolates displayed discordant genotypes. Isolate ULPS‐556 carried an avirulent allele of *Avr3c* and a virulent allele of *Avr4/6*, yet exhibited an avirulent phenotype towards *Rps3c*, *Rps4* and *Rps6*. Conversely, isolate ULPS‐778 possessed a virulent allele of *Avr3c* and an avirulent allele of *Avr4/6* and was virulent on soybean lines carrying *Rps3c*, *Rps4* and *Rps6*. These discordances suggest a recombination event between the two closely linked avirulence genes and indicate that virulence towards *Rps3c*, *Rps4* and *Rps6* is associated with *Avr3c* rather than *Avr4/6*. To validate these results, a modified version of the hypocotyl‐wound inoculation from Dorrance et al. (2008) was also performed on Williams, L92‐7857 (*Rps3c*), Haro34 (*Rps3c*), L85‐2352 (*Rps4*), L89‐1581 (*Rps6*), Harosoy, Haro4272 (*Rps4*) and Haro6272 (*Rps6*) (Figure 2). Results were consistent with those obtained in the hydroponic assays where ULPS‐556 is avirulent against the six resistant lines and ULPS‐778 is uniformly virulent against the same resistant lines, thus confirming the sole association of *Avr3c* with virulence towards *Rps3c*, *Rps4* and *Rps6*. To our knowledge, the haplotype of *Avr3c* and *Avr4/6* have always been reported as genetically linked owing to the close proximity of these loci. The recombination event described here represents a rare natural dissociation of the two genes and provides a unique opportunity to resolve their respective contribution to virulence.

**FIGURE 1:**
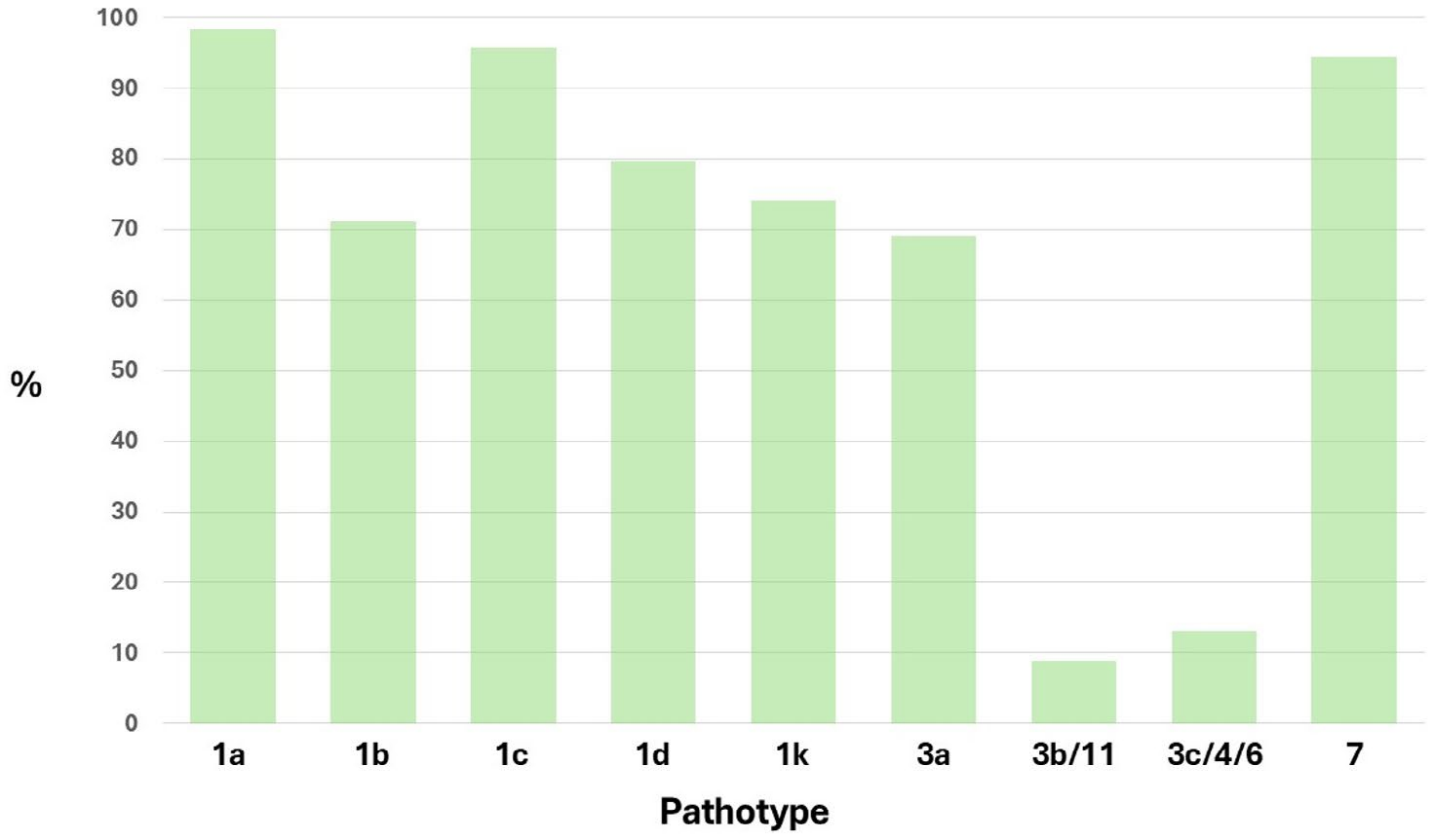
*Phytophthora sojae* pathotypes prevalence (%) in Canada. Pathotypes of 236 field isolates collected in all Canadian soybean‐producing provinces from 2018 to 2020. All isolates were retrieved from soil samples, isolated in pure culture and inoculated on a differential of soybean resistant lines carrying the *Rps* gene corresponding to each pathotype.

**FIGURE 2:**
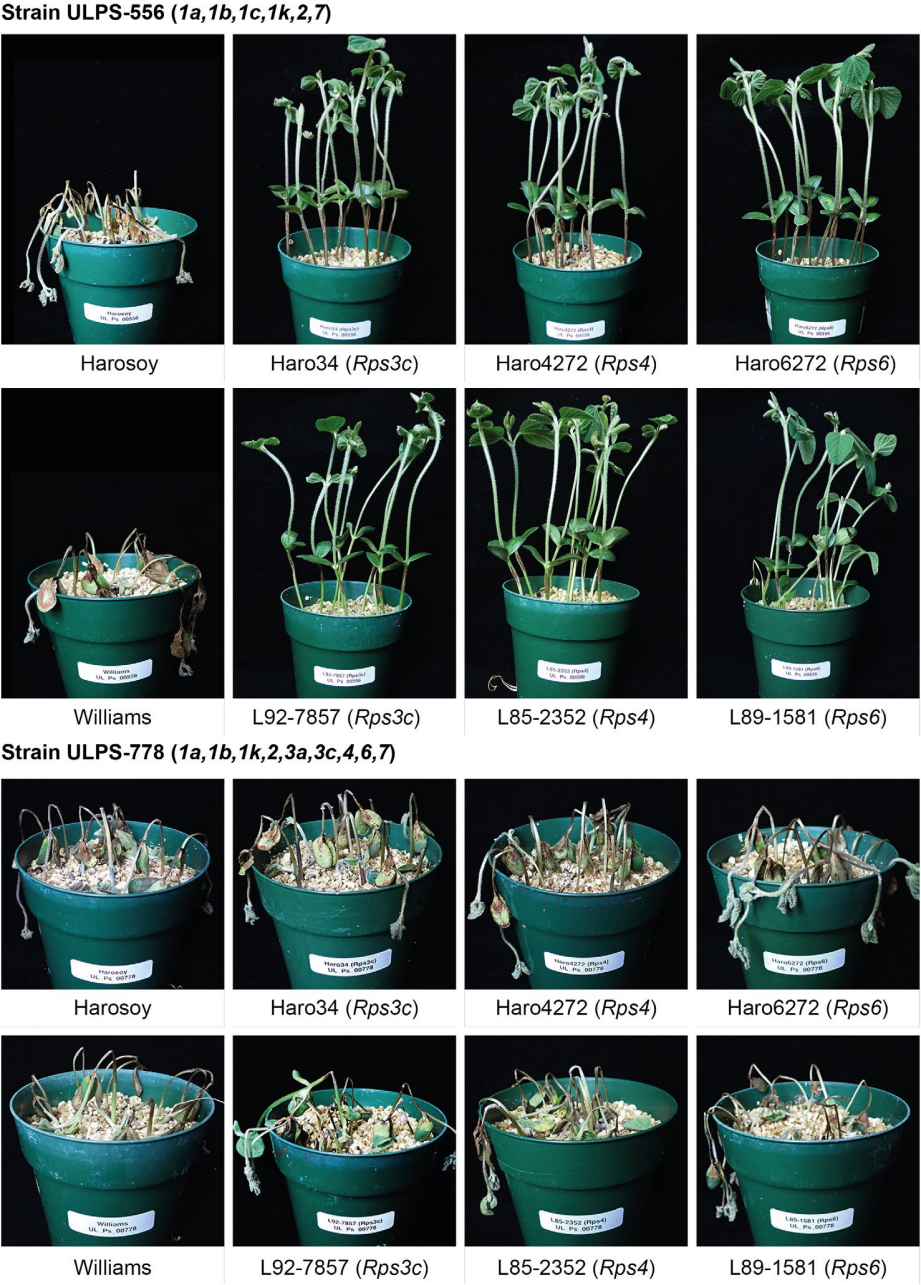
Phenotypic responses of *Rps3c*, *Rps4* and *Rps6* resistant lines. Phenotypes of L92‐7857 (*Rps3c*), Haro34 (*Rps3c*), L85‐2352 (*Rps4*), Haro4272 (*Rps4*), Haro6272 (*Rps6*), L89‐1581 (*Rps6*) and susceptible controls (Williams and Harosoy) using hypocotyl‐wound inoculation. Isolate ULPS‐556 carries the avirulent form of *Avr3c* and isolate ULPS‐778 carries the virulent one.

To investigate this rare recombination between effectors *Avr3c* and *Avr4/6* in isolates ULPS‐556 and ULPS‐778, sequencing on the MinIon from Oxford Nanopore Technologies and Illumina NovaSeq platforms were performed. For ULPS‐556 a coverage of 68× and 158× was obtained from long‐ and short‐read sequencing, respectively, whereas the sequencing of ULPS‐778 generated 46× and 150× of data. For each isolate, the local *de novo* assembly, using Flye v. 2.9.6 with default parameters (Kolmogorov et al. 2019), of the *Avr3c* and *Avr4/6* region on Chr5 produced gap‐free contigs of 175 and 180 kb including 50 kb up‐ and downstream of the genes. For comparative purposes, two other isolates without the recombination between the two effectors were also sequenced, one virulent to *Rps3c*, *Rps4* and *Rps6* (ULPS‐728) and one avirulent (ULPS‐733). Assemblies, from long and short reads, of 175 and 213 kb were respectively produced as virulent and avirulent references and annotated using the latest *P. sojae* genome assembly (Zhang et al. 2024). Global synteny, between physical position 343,700 and 518,850 of Chr5, was evaluated among the four isolates using MCScanX (Wang et al. 2012). Additionally, Illumina reads of isolates ULPS‐556 and ULPS‐778 were mapped on the virulent and avirulent references then the SNP variants were called using CLC Genomics Workbench (Qiagen). As shown in Figure 3, the isolate ULPS‐556 exhibits synteny with both the avirulent and virulent references. The haplotype surrounding *Avr4/6* shares a higher overall sequence identity with the virulent reference; however, the *Avr4/6* coding sequence itself is identical to that of the avirulent sequence, as no variants are detected. In contrast, the *Avr3c* region of ULPS‐556 is nearly identical to the avirulent reference with almost no SNPs observed. The opposite pattern is observed for isolate ULPS‐778. In this isolate, the *Avr4/6* region is highly similar to the avirulent reference, whereas the *Avr3c* region matches the virulent reference. As in ULPS‐556, no variants were detected within the *Avr4/6* coding region. The synteny analysis showed a higher structural conservation around *Avr4/6*; except for SNPs and a few small indels, no major structural rearrangements were found. However, a different scenario is detected around *Avr3c*. We observed a segmental duplication of a block of 33.7 kb, as reported by Dong et al. (2009), in the isolates avirulent to *Rps3c*, *Rps4* and *Rps6*. Three copies were found in our avirulent reference (ULPS‐733) but only two in ULPS‐556. The virulent *P. sojae* isolates also carried a repetitive segment, but one of 29.2 kb, with a haplotype distinct from the avirulent one. Altogether, greater variation is observed throughout the *Avr3c* region whereas *Avr4/6* is more conserved.

**FIGURE 3:**
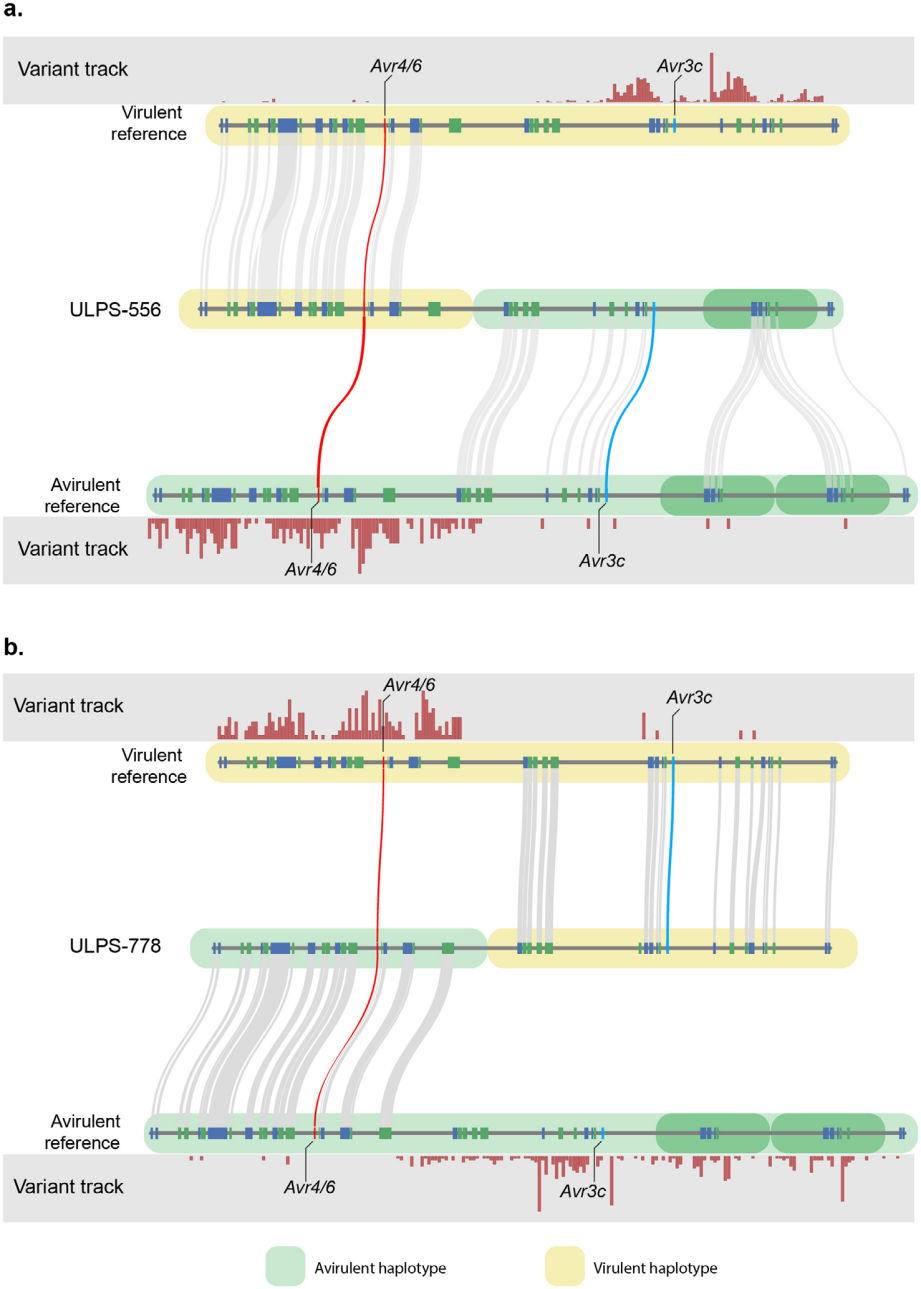
Synteny analysis at *Avr4/6* and *Avr3c* loci in two *Phytophthora sojae* field isolates. Synteny on Chr5 from positions 343,700 to 518,850 and variant coverage among the avirulent isolate ULPS‐556 (a) and the virulent isolate ULPS‐778 (b) compared to a virulent (ULPS‐728) and an avirulent (ULPS‐733) references. The green and yellow shaded areas represent the avirulent and virulent haplotypes whereas the darker green shaded area represents the segmental duplication. Green and blue boxes represent the predicted coding sequences of all genes and the grey shaded areas represent conserved syntenic blocks. SNP variant coverage is represented in the variant track.

The hypersensitive response (HR) is a classical proxy employed to determine the recognition of an avirulence gene by its corresponding resistance protein. Both *Avr3c* and *Avr4/6* have previously been reported to induce HR in soybean. Following the recent characterization and cloning of *Rps6* where *Avr3c* was shown to interact with the putative *Rps6*, we sought to determine whether *Avr4/6* could also trigger an HR in *Rps6*‐resistant lines. To address this question, a soybean protoplast cell‐death assay was developed following the methodology described in Asselin et al. (2026). Briefly, expression constructs containing the coding sequences of the functional allele of *Avr3c* and *Avr4/6* as well as one containing the coding sequence of the putative *Rps6* were generated. In addition, a luciferase reporter construct was included to quantify cell viability following co‐expression of the *Avr* and *R* gene constructs. Luciferase activity was normalized with the luciferase measures from an empty‐vector control, allowing cell death to be expressed as the reduction in viability relative to the *Avr* and *R* gene interaction (Table S3). The interaction between *MLA1* and *AVR*_*A1*_ (Saur et al. 2019) served as a positive control for HR induction. Recognition of *Rps6* corresponding effector is expected to trigger cell death, resulting in reduced luciferase activity relative to non‐interacting combinations. As shown in Figure 4, *Avr4/6* fails to elicit an HR when co‐expressed with *Rps6*, as relative luciferase activity was around 1, indicating no detectable loss of protoplast viability. In contrast, *Avr3c* triggered a strong *Rps6*‐dependent immune response, resulting in an average reduction of luciferase activity of 80%. This response was stronger than the interaction between the barley NLR *MLA1* and its corresponding effector *AVR*_*A1*_ from *Blumeria graminis*, which induces about 70% of reduction in luciferase activity. These results provide functional evidence that *Avr3c*, but not *Avr4/6*, is specifically recognized by *Rps6*.

**FIGURE 4:**
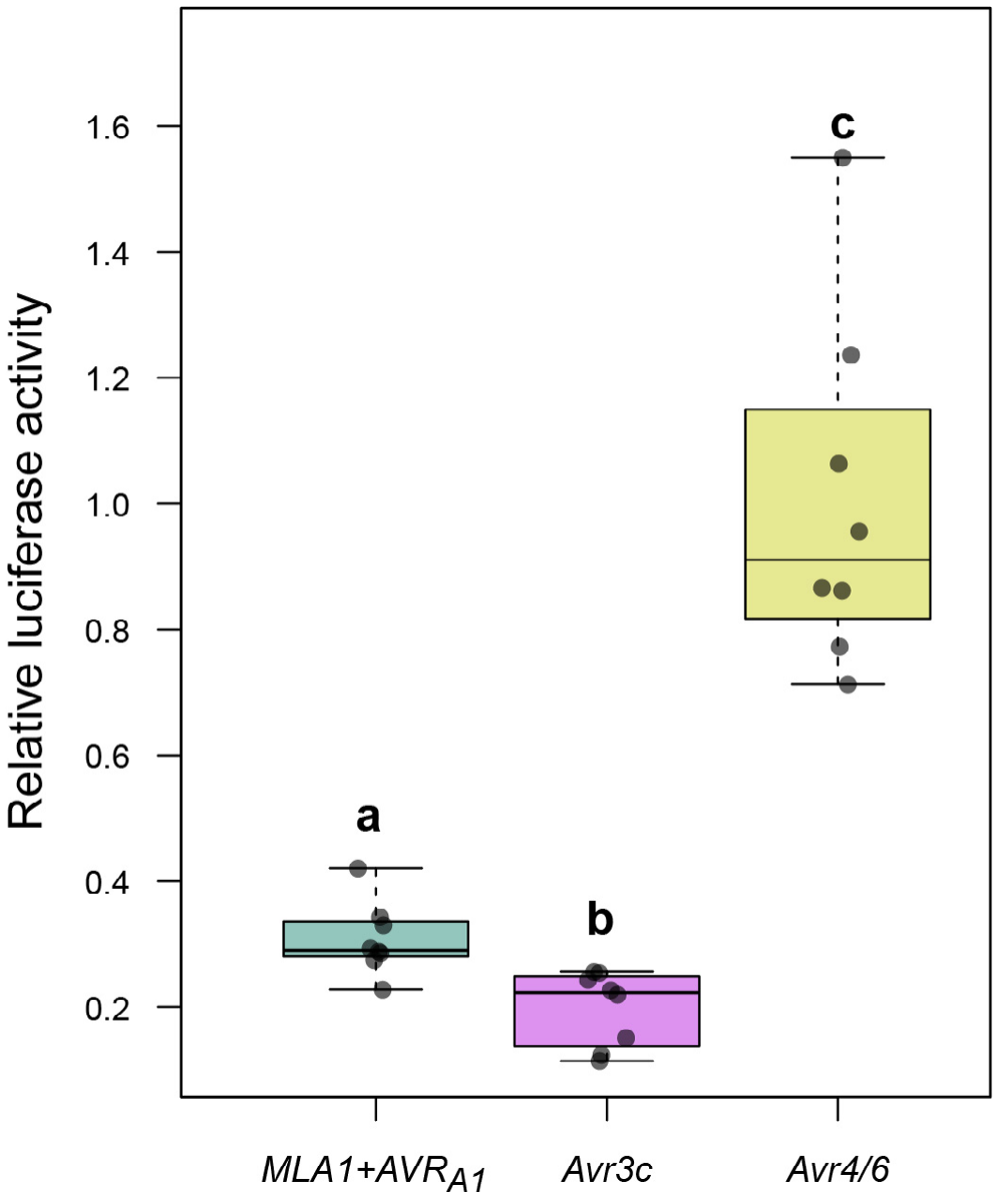
Boxplots of *Avr3c*‐ and *Avr4/6*‐dependent cell death response in soybean protoplasts expressing *Rps6*. Soybean protoplasts expressing the *Rps6* gene were co‐transfected with constructs encoding *Avr3c*, *Avr4/6* and luciferase. *MLA1* and *AVR*_*A1*_ were employed as a positive interaction control. Cell viability was quantified by relative luciferase activity, normalized to a luciferase control. Boxplots of eight biological replicates per treatment from three independent temporal replicates. Luciferase values were log‐transformed for statistical analysis to respect the homogeneity of variance but plotted untransformed. Differences among treatments were evaluated by Tukey's HSD test (*p* < 0.05) and marked by letters on the figure.

Plant pathogens exhibit tremendous intraspecific genetic variation, especially for genes under strong selection pressure to overcome host resistance. Among those, effectors and more specifically avirulence genes are offering some of the most diverse regions in their genomes. In this study, we found a rare recombination event in the *P. sojae* genome that allowed us to reassess the specificity of a major resistance gene, *Rps6*. While one avirulence gene, *Avr4/6*, has been previously associated with this source of resistance, we present evidence that the recognition process is rather linked with another one, *Avr3c*. Interestingly, based on a large natural population of *P. sojae*, we showed that alleles of *Avr3c* and *Avr4/6* were in perfect linkage in 234 out of 236 isolates. Original mapping by Whisson et al. (2004) positioned *Avr4/6* in a 4.3 cM region of Chr5. Assuming a 35.3 kb/cM recombination rate (Whisson et al. 2004), the 4.3 cM can possibly cover up to 151.8 kb, thereby including the *Avr3c* locus. We therefore suggest that Whisson et al. (2004) and Dong et al. (2009) both correctly associated the same locus with virulence towards *Rps6* resistant soybean lines.

*Phytophthora sojae* can evade recognition from its host by exploiting diverse strategies such as the mutation of key residues in the protein produced by its avirulence genes as in the case of *Avr1c*, *Avr3b* and *Avr3c* (Yang et al. 2019; Kong et al. 2015; Huang et al. 2019). Complete deletion of the genes *Avr1a*, *Avr1b*, *Avr1c* and *Avr1d* has also been described as another route for promoting virulence of the pathogen (Na et al. 2013; Qutob et al. 2009; Cui et al. 2012; Na et al. 2014). Finally, gene silencing by siRNAs was described for *Avr3a* but was also proposed for *Avr1a* and *Avr1c* (Qutob et al. 2013; Yang et al. 2019; Qutob et al. 2009). Considering all putative evasion strategies, *Avr4/6* is an oddity among avirulence genes. According to Dou et al. (2010) no sequence polymorphisms are present in its coding sequence and no transcriptional changes between virulent and avirulent strains are measured. This raises the question as to how can a protein avoid being recognized in some cases and not in others? Our results suggest that *Avr4/6* does not constitute the bona fide avirulence determinant underlying *Rps3c*‐, *Rps4*‐ and *Rps6*‐mediated resistance. Although, it was reported to induce cell death in *Rps4* and *Rps6* soybean lines, it is unclear whether the recognition was specific to this source of resistance or if the protein also induced immune responses in other resistant genotypes. In *Phytophthora* species, some conserved effectors are capable of inducing cell death without functioning as avirulence gene because the outcome of infection depends on the combined activities of hundreds of secreted proteins so that the effect of one effector is not always a good proxy for the whole plant response (Oh et al. 2024). Additional support for this interpretation comes from Fang and Tyler (2016) CRISPR‐Cas9 mutagenesis study in which *Avr4/6* was knocked‐out to assess its contribution to virulence towards *Rps4* and *Rps6*. Results showed that although an increase of virulence was found in the mutants, it was not comparable to their susceptible controls. The authors therefore concluded that ‘*Rps4* and *Rps6* loci make additional contributions to resistance other than through the recognition of *Avr4/6*’. Taken together with our genetic marker analysis, interactions assays and whole‐genome resequencing, these findings support *Avr3c* as the sole avirulence gene associated with recognition by *Rps3c*, *Rps4* and *Rps6*.

In this study, we provide new insights into the *P. sojae* virulence mechanisms and show that *Avr3c*, the corresponding effector of *Rps3c*, is also recognized by *Rps4* and *Rps6*. This result is coherent with the recent report that all three genes are in fact functionally redundant (Asselin et al. 2026) and establishes that *Avr3c* stands as the correct and sole avirulence gene for *Rps3c*, *Rps4* and *Rps6*. Given that *Rps6* is the only gene deployed commercially from the three *Rps*, we propose that *Avr3c* be renamed *Avr6* to better reflect its biological function and to simplify the nomenclature of the *Rps*–*Avr* interaction. Preserving *Rps6* effectiveness will require continued monitoring of pathogen populations and the strategic deployment of resistant cultivars where they are effective. By resolving the identity of the avirulence protein recognized by *Rps6*, this work provides a more reliable molecular target for *P. sojae* pathotyping and strengthens the foundation for resistance‐guided disease management in soybean.

## Author Contributions

**Caroline Labbé:** conceptualization, investigation, methodology, supervision, project administration, writing – review and editing, visualization, formal analysis, data curation. **Yanick Asselin:** conceptualization, investigation, writing – original draft, methodology, validation, formal analysis, data curation, software, visualization. **François Belzile:** writing – review and editing, supervision. **Richard Bélanger:** conceptualization, writing – review and editing, resources, project administration, supervision, funding acquisition. **Vanessa Tremblay:** data curation, formal analysis. **Adèle Deshaies:** formal analysis, data curation.

## Funding

Research supported by Genome Canada (10.13039/100008762), Génome Québec (10.13039/100013062) and Canada Research Chairs (10.13039/501100001804).

## Conflicts of Interest

The authors declare no conflicts of interest.

## Supporting information


**Figure S1:** Amino acid alignment of *Avr3c* and *Avr4/6* protein sequence. (a) Protein sequences alignment of all alleles of *Avr3c* as reported by Huang et al. (2019) with their corresponding phenotype on *Rps3c*, *Rps4* and *Rps6* resistant lines. Only mutations and their positions are shown including the key residue associated with virulence, highlighted in red. The alignment was obtained from CLC Genomics Workbench. (b) Amino acid sequence of *Avr4/6* from the reference genome P6497. Huang et al. (2019): Natural allelic variations provide insights into host adaptation of *Phytophthora* avirulence effector PsAvr3c. *New Phytologist*, 221, 1010–1022.

**Table S1:** *Phytophthora* *sojae* field isolates phenotypes and genotypes for *Avr3c* and *Avr4/6*.

**Table S2:** PCR primers employed for genotyping the *Phytophthora sojae* population.

**Table S3:** Relative luciferase measures from the cell death assay.

## Data Availability

The datasets generated during the current study are available in the NCBI SRA repository, under the bioproject PRJNA1327418. The soybean accessions are available at the USDA germplasm collection (https://npgsweb.ars‐grin.gov/).
